# Utility of Braden Scale Nutrition Subscale Ratings as an Indicator of Dietary Intake and Weight Outcomes among Nursing Home Residents at Risk for Pressure Ulcers

**DOI:** 10.3390/healthcare3040879

**Published:** 2015-09-24

**Authors:** Susan Kennerly, Lisa Boss, Tracey L. Yap, Melissa Batchelor-Murphy, Susan D. Horn, Ryan Barrett, Nancy Bergstrom

**Affiliations:** 1 School of Nursing, University of North Carolina at Charlotte, Charlotte, NC 28223, USA; 2 School of Nursing, UT Health Houston, Houston, TX 77030, USA; E-Mails: lisa.boss@uth.tmc.edu (L.B.); nancy.bergstrom@uth.tmc.edu (N.B.); 3 School of Nursing, Duke University, Durham, NC 27710, USA; E-Mails: tracey.yap@duke.edu (T.L.Y.); melissa.batchelor-murphy@dm.duke.edu (M.B.-M.); 4 School of Medicine, University of Utah, Salt Lake City, UT 84108, USA; E-Mail: susan.horn@hsc.utah.edu; 5 International Severity Information Systems and the Institute for Clinical Outcomes Research, Salt Lake City, UT 84102, USA; E-Mail: ryanscottbarrett@gmail.com

**Keywords:** nutrition, nutritional risk, pressure ulcers, Braden Scale, nursing home, TURN Study

## Abstract

The Braden Scale for Pressure Sore Risk^©^ is a screening tool to determine overall risk of pressure ulcer development and estimate severity of specific risk factors for individual residents. Nurses often use the Braden nutrition subscale to screen nursing home (NH) residents for nutritional risk, and then recommend a more comprehensive nutritional assessment as indicated. Secondary data analysis from the Turn for Ulcer ReductioN (TURN) study’s investigation of U.S. and Canadian NH residents (*n* = 690) considered at moderate or high pressure ulcer (PrU) risk was used to evaluate the subscale’s utility for identifying nutritional intake risk factors. Associations were examined between Braden Nutritional Risk subscale screening, dietary intake (mean % meal intake and by meal timing, mean number of protein servings, protein sources, % intake of supplements and snacks), weight outcomes, and new PrU incidence. Of moderate and high PrU risk residents, 61.9% and 59.2% ate a mean meal % of <75. Fewer than 18% overall ate <50% of meals or refused meals. No significant differences were observed in weight differences by nutrition subscale risk or in mean number protein servings per meal (1.4 (SD = 0.58) *versus* 1.3 (SD = 0.53)) for moderate *versus* high PrU risk residents. The nutrition subscale approximates subsequent estimated dietary intake and can provide insight into meal intake patterns for those at either moderate or high PrU risk. Findings support the Braden Scale’s use as a preliminary screening method to identify focused areas for potential intervention.

## 1. Introduction

Compromised nutritional status has been linked to pressure ulcer (PrU) development for at least four decades, yet it remains an unresolved risk [1,2,3,4,5,6,7,8,9,10,11,12]. Early observational studies explored the relationship between factors, such as serum albumin, lower hemoglobin, lower dietary calories, and protein intake, with PrU incidence [1,2]. More recent studies expanded our understanding of PrU development by examining the link between PrUs and individual demographic characteristics, including body mass index (BMI), unintended weight loss, and medical diagnoses [3,4]. Few studies have prospectively observed dietary intake, nutritional risk status, and PrU development to draw conclusions about thresholds of overall intake related to PrU incidence and such studies are time and resource intense [5,6,7,8]. Much remains to be learned about how to best identify in routine care, nutritional risk as part of PrU prevention in nursing home (NH) residents.

A common approach to determine PrU risk upon NH admission is the use of the Braden Scale for Pressure Sore Risk^©^ (Prevention Plus, LLC, Omaha, NE, USA) [13] (hereafter Braden Scale). The Braden scale serves a dual role as a screening tool: (1) to determine a resident’s overall risk for PrU development; and (2) for estimation of severity of the most significant risk factors for the purpose of guiding care planning. This paper focuses on nutritional risk based on the nutrition subscale, one of the Braden Scale’s six domains used to assess rate overall PrU risk. The nutrition subscale is used by some NHs to screen for common factors leading to nutritional risk, but is not to be considered a comprehensive nutritional assessment. It is often the first method used upon NH admission to screen for adequacy of dietary intake as a potential contributor to overall PrU risk and to signal nursing and dietary staff of the need for a more comprehensive nutritional assessment and potential intervention. We believe that in order to establish successful nutritional interventions consistent with PrU prevention the associations between initial Braden Scale nutrition subscale (hereafter nutrition subscale) screening, dietary intake, and individual outcomes (e.g., BMI and weight change) must be clarified. This manuscript reports on evaluation of the utility of the nutrition subscale as a basis for identifying nutritional intake risk factors through secondary analysis of data from the TURN study’s [14] investigation of long-stay NH residents who were rated as moderate or high-risk for PrU development. The aims are to: (1) determine if licensed nurse nutritional risk ratings using the nutrition subscale differ significantly from observed risk in relation to intake of meals (breakfast, lunch, dinner), protein meal servings, or dietary supplements; and (2) determine the utility of staff nutritional risk ratings using the nutrition subscale score as an indicator of observed dietary adequacy outcomes, such as intake and weight change (loss, gain).

## 2. Experimental Section

### 2.1. Design

The TURN Study [14], a multisite clinical trial, was conducted to determine the optimal repositioning intervals (2, 3, or 4 h) for PrU prevention in nursing home residents observed for a 3 week study period while cared for on high-density foam mattresses. Nutritional risk, dietary intake, and PrU risk assessed during the TURN study are the focus of this evaluation of the utility of the Braden subscale for preliminary nutritional screening.

### 2.2. Setting and Participants

TURN study [14] participants were residents recruited from NHs in the U.S. (*n* = 20) and Canada (*n* = 7). Study participants were ≥65 years, without PrUs at the beginning of the study, and were stratified according to PrU risk as moderate (scores of 13–14) or high (scores of 10–12) according to the Braden Scale. Those residents deemed to be at low PrU risk or severe PrU risk were excluded from TURN study participation, because the study investigated differences in PrU incidence with repositioning at 2-, 3-, and 4-h intervals among moderate and high risk residents for whom regular repositioning at 2-h intervals to relieve pressure is the standard of care.

Before agreeing to participate in this study, each resident and/or legal representative was informed of the purpose, procedures, risks, and potential benefits of the study through a statement of informed consent. The Institutional Review Boards (IRB) of UT Health Houston, the University of Toronto, and a hospital associated with one of the NHs approved the study. The remaining U.S. NHs obtained Federal-Wide Assurance in relation to recognition of IRB acceptance prior to data collection.

### 2.3. Framework for Nutritional Risk Evaluation

**Figure 1:**
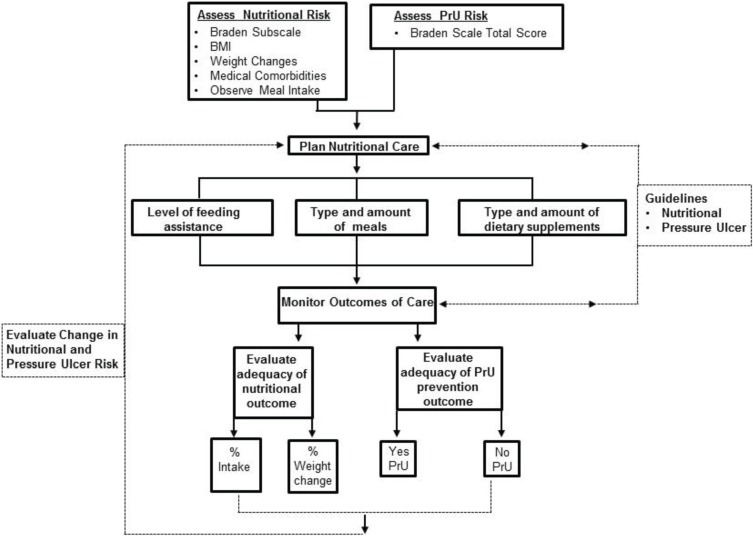
Framework for Nutritional Risk Evaluation and Care Planning in Relation to high pressure ulcer (PrU) Prevention.

This investigation of the utility of the nutrition subscale in relation to nutritional risk, dietary intake, and PrU risk was guided by the researcher developed framework for nutritional risk evaluation and care planning in relation to PrU prevention outcome (Figure 1). Risk assessment incorporated screening using the Braden Scale to ascertain overall risk for PrU development and nutritional risk status along with routine observations of BMI, change in weight, meal intake, and medical comorbidities, such as impaired mobility and cognition that might influence the resident’s ability to intake and use nutrients. Nutritional risk is defined as the assessment rating of a resident’s usual food intake pattern (usually over several days) according to the nutrition subscale categories 1—Very Poor, 2—Probably Inadequate, 3—Adequate, and 4—Excellent. These screening results were used along with the NH’s customary standards for nutritional care and PrU prevention and informed NH staff’s development of a nutritional plan of care. Common information points gathered to inform basic care planning included amount (%) of meal intake, protein sources, type and amount of dietary supplements, and level of feeding assistance required by each resident. A specific dietary plan was developed by the dieticians associated with each NH according to facility interpretation of regulatory requirements and best practices. Nutritional and PrU prevention guidelines also informed staff decisions about how to best monitor outcomes of care and which outcomes should be evaluated. Outcome evaluation in this investigation included a determination of the adequacy of nutritional outcomes (% intake, % weight change) and adequacy of PrU prevention judged by PrU incidence. The results of outcome evaluation provide the basis for making further refinements in the plan for nutritional and PrU prevention care.

### 2.4. Measurement

Data collection instruments included the Braden Scale and investigator developed tools that captured demographic (age, gender, race, diagnoses), dietary meal intake (% meal intake, # protein servings), and supplements (# snacks, # dietary supplements, and % consumed), brief changes (# wet and soiled changes), and resident’s NH.

#### 2.4.1. Braden Scale for Pressure Sore Risk

Extensively used in acute, home, and long-term care settings, the Braden Scale is intended to be part of a comprehensive clinical assessment to predict PrU risk. Overall PrU risk is determined according to six subscales, including sensory perception, skin moisture, activity, mobility, nutrition, and friction/shear [15,16,17]. For each subscale, a score is assigned ranging from one to four, indicating the level of capability/limitation related to the respective risk factor with descriptors defining the level of impairment. All items are summed and the score, ranging from 6 to 23, indicates overall PrU risk. A cut-off score of ≤18 identifies at-risk residents. Suggested time points for administration of the instrument differ based on care setting; however, it should always be completed upon admission and repeated within 24 hours and weekly for 4 weeks, as well as when a change in resident condition occurs [15]. Widely tested in various populations, inter-rater reliability is acceptable at 0.83–0.99, Cronbach’s α ranges from 0.83 to 0.99, sensitivity ranges from 83% to 100%, and specificity ranges from 64% to 90% [17,18]. In this study, licensed nurses screened each resident for PrU risk using the Braden Scale, inclusive of the nutritional risk subscale on admission, and weekly over a three week period.

#### 2.4.2. Nutritional Risk Ratings

The nutrition subscale of the Braden Scale is an assessment of the resident’s usual food intake pattern (usually over several days) and possible scores range from one to four [19]. Overall the subscale addresses dietary intake, number of protein servings, supplements and snacks, and tube feeding. A score of one, or Very Poor, indicates that the resident never eats a complete meal, rarely eats more than 1/3 of any food offered, eats ≤2 servings of protein, takes fluids poorly, does not take a liquid dietary supplement, takes nothing by mouth, and/or is maintained on clear liquids or intravenous fluids for more than five days. A score of two, or Probably Inadequate, indicates the resident rarely eats a meal and generally eats about ½ of food offered, protein intake is 3 servings per day, occasionally takes a liquid supplement, or received less than optimum amount of liquid diet or tube feeding [16]. A score of three, or Adequate, indicates the resident eats over half of most meals, eats a total of four servings of protein per day, occasionally may refuse a meal, but will take a liquid supplement when offered, or is tube fed or on prescribed total parenteral nutrition. Finally, a score of four, or Excellent, indicates the resident eats most of every meal, never refuses a meal, usually eats a total of four or more servings of protein per day, occasionally eats between meals, or does not require liquid supplementation [19].

#### 2.4.3. Dietary Intake

Dietary intake was measured through estimation of overall meal intake, protein meal intake, and dietary supplements. Throughout the study, dietary intake was estimated daily by certified nursing assistants (CNAs) according to: Percent (%) of each meal eaten, number of servings of protein eaten at each meal, sources of dietary protein, dietary supplements, and snacks.

##### Overall Meal Intake

The % intake at breakfast, lunch, and dinner, was documented by CNAs on TURN study data collection forms. For each meal, the CNA estimated the % eaten according to the following categories: 100%, 76%–99%, 51%–75%, 26%–50%, 1%–25%, refused, NPO, or tube feeding. The % intake for each meal was summed to obtain the % of the total estimated intake for each day and grouped into the following four categories for analysis: (<50% poor, 50% inadequate, >50% but <75% adequate, >75% excellent). While, the percent dietary intake is a highly subjective estimate of intake, it has been demonstrated previously to have moderate correlation with observed dietary intake, with under rather than over estimating intake [3]. Further, the estimation method offered a basis for intake comparison across residents, while not increasing the care burden of staff that is typically associated with more exacting measures, such as used in an early study by Bergstrom and Braden [7].

##### Protein Meal Intake

Published evidence exists associating dietary intake and PrUs, and it is generally accepted that current dietary intake of protein is a more important predictor of PrUs than blood or serum (e.g., hemoglobin, albumin) and anthropometric measures [4]. For each meal, CNAs were instructed to document the number of protein servings on TURN study forms according to the following categories: egg (1), dairy/milk (1 cup), cheese (1 oz.), fish/meat (3 oz.), luncheon meat, beans/legumes, soy protein/tofu, nuts/peanut butter, or protein powder. Total number of protein servings at each meal were then placed into the following categories for analysis: <2 = Very Poor; 3 = Probably Inadequate; 4 = Adequate, >4 = Excellent).

##### Dietary Supplement Intake

For any resident receiving liquid supplements or snacks, the CNAs were instructed to estimate the % consumed with each offering of supplement or snack and document on TURN study forms in the following categories: 100%, 76%–99%, 51%–75%, 26%–50%, 1%–25%, or refused. Total % liquid supplement or snack consumed at each meal was then placed into the following categories for analysis: supplement or snack not consumed = Very Poor; occasionally take a supplement or snack = Probably Inadequate; usually take a supplement or snack = Adequate, requires no supplement or snack = Excellent. Nutrition provided by supplements was estimated, since supplement intake goals often fail due to the disruption of feeding, feeding tolerance issues, and interruption when residents are off the unit or out of the facility [20].

#### 2.4.4. Dietary Adequacy

Change in weight (loss, gain) served as observed outcome indicators of dietary adequacy. CNAs were instructed to measure resident body weight per facility protocol. Timing may have varied somewhat by facility, but weights were typically measured upon admission and weekly thereafter. Weights were recorded in the resident’s medical record with transcription onto TURN study forms. The TURN study data collector was responsible for reviewing the data and determining which of the following categories corresponded with the resident’s weight loss/gain: 5%–10% loss/gain in last 30 days or >10% loss/gain during the last 30 days.

### 2.5. Data Collection/Methods/Procedures

Resident participants were randomized by risk level (moderate *versus* high) to a repositioning schedule (2-, 3-, or 4-h), repositioned and monitored, and skin areas (coccyx/sacrum, trochanter, heels) were assessed at each turn by CNAs during repositioning and weekly by a licensed nurse assessor blinded to turning frequency. Variables related to PrU development were monitored throughout the 3-week study with specific observations related to dietary intake, the focus of this report. Training was provided for nurse assessors to standardize use of the Braden Scale and included videos, vignettes, observation, and nurse assessors to establish interrater reliability for Braden scoring (*r* = 0.93). All CNAs completed required training regarding repositioning, estimation of dietary intake, and intake and repositioning related study documentation [14]. Blinding of licensed staff performing risk assessments to CNA documentation was achieved by concealment of documentation in a closed folder and risk assessments being performed for residents on a unit to which the licensed staff were not assigned. Further details about study methods and findings are reported elsewhere [14].

#### Data Management and Analysis

Mean meal intake (%) by meal timing (breakfast, lunch, dinner), mean number of protein servings, and mean protein sources, the estimated % of intake of supplements and snacks, and incidence of a new PrU were monitored for participating residents across the 3 week study period at each NH. Dietary intake data were transformed from a meal intake range to a resident level intake % for analysis purposes. Intake ranges were documented by CNAs for each meal (e.g., 1%–25%, 26%–50%, *etc.*). To calculate mean resident intake levels, midpoints were used (e.g., 1%–25% became 12.5%, 26%–50% became 38%); NPO and refused were assigned values of 0%, while tube feeding episodes were excluded from calculations. Means were calculated in the usual manner, using a numerator and denominator of number of present meal observations (*i.e.*, missing meal documentations were not assigned a 0%). Tube-fed residents were excluded from analysis of dietary intake, since specific tube feeding intake was not recorded and few residents received tube feedings.

Percentage of meal consumed was multiplied by number of protein servings prior to calculation of overall mean (e.g., if the record showed a resident consumed one serving of egg and one serving of cheese for breakfast, but only ate 12.5% of the meal, the protein servings for that meal would be 0.125 × (1 + 1) = 0.25); this allowed for a more specific estimate of protein consumed at each meal. Mean liquid supplement and snack consumed were calculated in two ways. The first used a denominator of number of times in which the supplement/snack was actually consumed (*i.e.*, excluding those meals where the supplement/snack was not consumed from the denominator); the second included those meals in the denominator.

Repeated measures of Braden scale and subscale scores were collected at admission and weekly. If there was variation in scores from week to week, the most commonly reported Braden score was used in the resident level analyses.

Descriptive analyses included examination of frequencies for categorical measures and means and standard deviations for continuous measures. Correlations were examined between Braden Scale and subscale risk categories and within risk categories according to study measures of dietary intake and dietary adequacy. The strength of association between variables was estimated using Spearman *rho* correlation coefficients. Satterthwaite two sample t-tests or paired comparisons of means with analysis of variance and Duncan’s Multiple Range Test were used for continuous variables. SAS version 9.2 [21] was used to perform statistical analyses. Sample size was determined adequate to support stratification of variables by PrU risk and nutritional risk categories and detect differences at a two-tailed alpha of 0.05 and power of 0.80.

## 3. Results

Resident participants in the TURN Study (*n* = 942) who were Asian (*n* = 101) or short stay (*n* = 128) were excluded from this analysis, which included 721 participants who resided in nursing facilities >90 days. Short stay residents are likely different than long stay residents due to recent illnesses leading to transfer from hospital to nursing homes or substantial changes in condition requiring moving from home to nursing home; recent illness and relocation stress may influence dietary intake and inflammatory responses related to nutritional status [22]. Asian residents were significantly different in body mass index and dietary intake from other residents (*p* < 0.001, *p* = 0.009, respectively), were removed from the analysis, and will be reported elsewhere. After preliminary analysis, 23 (3.19%) of the 721 subjects were found to have tube feedings during all or part of the study. Although enteral nutrition has many favorable effects, it commonly fails to provide adequate energy requirements for a variety of reasons, including improper feeding tube location, frequent interruption, gastrointestinal intolerance, feeding tube problems, and underprescription by healthcare providers [20]. For these reasons, tube fed residents were not included in the analysis resulting in 698 residents whose dietary intake was observed for 3 to 42 days (mean = 20.17, median = 21, SD = 3.969). Of the remaining 698 residents, 8 were excluded from the analysis due to missing Braden Scale data, leaving a final sample size of *n* = 690.

### 3.1. Resident Participant Characteristics

Descriptive analyses, correlations, and paired comparisons were used to examine characteristics for residents in this study (*n* = 690) including gender, age, BMI, and primary medical diagnoses (Table 1), which are characteristics often considered to be potential contributors in PrU development. Residents were categorized according to Braden Scale PrU Risk Score moderate (*n* = 462) and high risk (*n* = 228) categories and according to staff ratings of Braden Scale nutrition subscale risk categories. All 4 nutrition subscale risk categories were represented among the 690 residents: category 1—Very Poor, (*n* = 25), category 2—Probably Inadequate (*n* = 214), category 3—Adequate (*n* = 403), and category 4—Excellent (*n* = 48). Mean ages ranged from 80.9 years in category 4 to 87.5 years in category 1 and residents were predominately white females (72%). Nutrition subscale category 1 *versus* category 4 residents had significantly lower BMI (mean 22.3 *versus* 27.8 (Kg/m^2^), higher percentage of females (84% *versus* 70.8%), and lower total Braden Scores (11.8 *versus* 12.9).

Dementia was the most frequently occurring diagnosis among nutrition subscale risk category 1 residents, but was not significantly more frequent than in other nutritional categories. Cerebrovascular disease and diabetes mellitus occurred more frequently in subscale category 4 residents (39.6% and 35.4%, respectively). Musculoskeletal and thyroid disorders, however, were significantly more common in subscale category 1 residents when compared with other nutrition subscale risk categories (72%, *p* = 0.015; 36%, *p* = 0.018, respectively). A diagnosis of nutritional disorder occurred most frequently among category 1 residents (4%). No residents were diagnosed with delirium. Five residents (0.72%) had >10% weight loss during the study; 12 (1.7%) had 5%–10% weight loss; 11 residents (1.6%) had 5%–10% weight gain; the overwhelming majority (662; 95.9%) did not have any weight loss/gain during the study period.

**Table 1:** Resident Participant Characteristics Gender, Age, BMI, Diagnoses, and Braden Scale Score Grouped According to Braden Nutrition Subscale Risk Categories (*n* = 690).

Resident Characteristic	Nutrition 1 Risk: Very Poor (*n* = 25, 4%)	Nutrition 2 Risk: Probably Adequate (*n* = 214, 31%)	Nutrition 3 Risk: Adequate (*n* = 403, 58%)	Nutrition 4 Risk: Excellent (*n* = 48, 7%)	*p* Value	Test
	Mean, #, or %	Mean, #, or %	Mean, #, or %	Mean, #, or %		
**Gender:**						
Female	84%	86.5%	76.9%	70.8%	0.014 *	Chi-Square
Male	12%	7.5%	5.7%	6.3%		
**Age**	87.3	87.5	84.9	80.9	<0.001 **	ANOVA + Duncan lines
**BMI**	22.3	23.1	26.3	27.8	<0.001 **	ANOVA + Duncan lines
**Diagnoses:**						
Cerebrovascular Disease	28%	32.7%	39.4%	39.6%	0.905	Chi-Square
Dementia	84%	80.1%	75.1%	83.3%	0.293	Chi-Square
Diabetes Mellitus	12%	20.9%	26.4%	35.4%	0.059	Chi-Square
Musculoskeletal Disorder	72%	61.1%	52.6%	41.7%	0.015 *	Chi-Square
Nutritional Diagnosis	4%	0%	1.5%	2.1%	0.171	Chi-Square
Thyroid Disorder	36%	22.8%	16.2%	12.5%	0.018 *	Chi-Square
**Race:**						
African American	12%	7.5%	5.7%	6.3%	0.619	Chi-Square
Caucasian	84%	89.7%	90.8%	93.8%		
Hispanic	4%	1.4%	3%	0%		
Other	0%	1.4%	0.5%	0%		
**Braden Scale (PrU Risk)**	11.8	12.6	13.1	12.9	<0.001 **	ANOVA + Duncan lines

* significant at *p* < 0.05; ** significant at *p* < 0.001.

Of the 690 residents studied, 10 (1.5%) developed a PrU. Mean age for these residents was 88.7 years, and mean BMI was 26.7. Similar to the overall sample, all were female and 90% were white. Diagnoses reported included cardiovascular disease (90%), dementia (80%), musculoskeletal disease (70%), diabetes mellitus (40%), cerebrovascular disease (10%), and thyroid disorder (10%). A nutritional diagnosis was only reported in 10%. Mean Braden Scale score was 13, indicating moderate risk for PrU development. Mean Braden Scale subscale scores for sensory perception, moisture, activity, and mobility ranged from 2.0 to 2.7, and the mean friction shear subscale score was 1.44. Most residents developing a PrU were bed bathed (60%) on the majority of days in the week (M = 4.56 baths per week). Help eating was required by most residents (70%) with a new PrU and 70% ate 100% of meals. The mean protein serving per meal was 1.22 and most residents favored meat (0.31) and milk (0.45) as protein sources. In addition, 90% of these residents received a liquid supplement on at least 1 day. On 49% of days, however, residents consumed 63% of the liquid supplement. There was no reported weight loss or gain for these residents.

### 3.2. Dietary Intake Findings

Dietary intake of 690 residents was monitored by CNAs at the resident’s respective NH. Results of analyses for % of each meal eaten, number of servings of protein eaten at each meal, sources of dietary protein, dietary supplements, and snacks are summarized individually and in relation to Braden Scale PrU risk and Braden nutrition subscale risk categories.

#### 3.2.1. Meal Intake

CNAs reported that 59.2% of high PrU risk and 61.9% of moderate PrU risk residents ate a mean of ≤75% of most meals. There was no significant difference in dietary intake categories between risk levels. Fewer than 18% of residents in both groups were reported to eat less than 50% of meals or refuse meals most days. The mean meal intake estimated by using the midpoint of the reported range for each individual for each meal is 75% (±SD) for high risk and 77% (±SD) for moderate risk residents. The mean intake across meals (breakfast, lunch, and dinner) was highest for breakfast and lower, but nearly identical for lunch and dinner and was not significantly different by meal or PrU risk level. Thirteen residents were reported to be NPO or refuse feedings; the mean percent intake across the study period for these residents ranged from 8.3% to 48%. Table 2 summarizes mean % of meal intake and Braden Scale PrU Risk Score according to the 4 nutrition subscale risk categories.

The mean % of meal intake varied by the 4 categories of nutritional risk ratings with increases in a stepwise fashion across the categories from Very Poor to Excellent. The range in % mean meal intake was as low as 8.33 in category 2 and as high as 100% in categories 2, 3, and 4. Each of the nutritional risk categories was comprised of residents from both moderate and high PrU risk categories with mean PrU risk score ranging from 11.80 to 13.06. However, average meal intake consumed was not significantly correlated (*rho* = 0.57, *p* = 0.138, *n* = 690) with being at moderate or high Braden Scale PrU risk. The mean Braden Scale PrU risk for those residents (*n* = 10) who developed a PrU was 13 (moderate risk).

**Table 2:** Mean and Range of % Meal Intake and Braden Scale PrU Risk Score Grouped According to Braden Nutrition Subscale Risk Categories (*n* = 690).

Braden Nutrition Risk Subscale Category	Mean % (Range) Meal Intake	Braden Scale PrU Risk Mean (Range) Score
1 = Very poor	48.67% (19.33 to 88.03)	11.80 (10 to 14)
2 = Probably Adequate	65.11% (8.33 to 100)	12.55 (10 to 14)
3 = Adequate	82.04% (19.11 to 100)	13.06 (10 to 14)
4 = Excellent	92.80% (21.70 to 100)	12.85 (10 to 14)

Percent of meal consumed by males and females ranged from 8.33 to 100%. Males with a Braden nutritional risk of 3—Adequate, had a significantly greater mean % of meal consumption (Male = 86.84; Female = 80.59) (Satterthwaite *t*-test, *t* = −3.28, *p* = 0.0013). Analysis of variance showed no significant differences by race (African American, Hispanic, and Caucasian) for Braden Nutrition category 1—Very Poor or 2—Probably Inadequate, however, significant differences observed in relation to categories 3—Adequate and 4—Excellent. Mean % meal intake for Caucasians (94.02) in the excellent category is significantly greater (F = 9.78, *p* < 0.0031) than for African Americans (72.56) with no Hispanics represented. Mean % meal intake differed significantly (F = 4.11, *p* = 0.0069) between races for residents in category 3—Adequate, with Hispanics (69.8%) and African Americans (74.8%) having significantly lower intake than residents in the other category (Excellent 97.4%). A diagnosis for a nutritional problem was only present for 8 (1.16%) residents with 6 of these observed as being in the nutritional risk of 3—Adequate category. There was no significant difference (*t* = 0.87, *p* = 0.383) in average meal intake for residents with a nutritional diagnosis. Mean age of those whose nutritional risk was deemed to be very poor to probably inadequate was 87; however, age was not significantly correlated (*p* ≥ 0.05) with average meal intake of residents in these nutritional risk categories.

Braden Scale Activity and Mobility subscale scores were examined in relation to Braden nutritional risk and % meal intake. A low, yet significant correlation (*rho* = 0.107, *p* = 0.03) was observed between Braden Activity subscale score and average estimated meal intake (mean 82.04%, 19.11% to 100%) for the group of residents with nutrition risk rating of 3—Adequate (*n* = 403). Staff rating of nutritional risk corresponded more closely with estimated meal intake for residents whose risk rating was adequate than for the other 3 risk categories. A moderate negative, significant correlation (*rho* = −0.42, *p* = 0.04) was observed between Braden Mobility subscale score (mean = 2.12/very limited, range 1 to 3) and average estimated meal intake (mean 48.67%, 19.33% to 88.03%) for the group of residents with nutritional risk rating of 1—Very Poor (*n* = 25). Staff nutrition subscale risk rating of residents in this group was based on their screening observation that meals were never consumed in their entirety and rarely was 33% intake reached. All of the residents in this nutritional risk group (*n* = 25) experienced a substantial degree of limitation in their ability to change and control body position, especially to do so independently, which may have contributed to a lower % of meal intake. Additionally, 21 (84%) of residents in the “very poor” nutrition subscale risk category had a diagnosis of dementia and may have been more easily distractible, another factor that may help to explain why achieving a higher % of meal intake was a challenge. The inverse nature of the relationship may be explained by the possibility for residents with fewer mobility limitations to receive less feeding assistance (*n* = 5 requiring setup only; *n* = 4 eat independently) suggesting that % of intake in such a situation may be potentially compromised. Data regarding dental status and swallowing function of residents were not collected, thus limiting our ability to explain their potential impact on dietary intake.

#### 3.2.2. Protein Servings and Protein Sources

The mean number of servings and the source of protein reported by CNAs at each meal was adjusted by the percent of the meal eaten. Overall, the estimated mean number of protein servings per meal was 1.3 (±SD = 0.53) *versus* 1.4 (±SD = 0.58) for high *versus* moderate PrU risk residents and was not significantly different. Sources of protein recorded at each meal varied, but the predominant sources of protein for both moderate and high PrU risk groups were meat, milk, and eggs. Other sources of protein were highly variable. The use of protein supplements added to food was infrequently reported. Nutrition subscale risk categories and the corresponding number of protein servings and protein sources are presented in Table 3. Number of protein servings per meal increased across the nutritional risk categories. When the mean % intake reached 92.8% for residents rated as being nutrition subscale risk category 4—Excellent, the mean # of protein servings per meal times 3 meals per day yields 5.56, which is consistent with the 4 or more servings recommended per day as part of a standard diet. For residents comprising the Probably Adequate nutrition subscale risk category, the mean # of protein servings per day was 3.36. As with mean % of intake, the # of protein servings per meal (0.71 or 2.13 per day) was lowest for those residents in the Very Poor nutrition subscale risk category.

**Table 3:** Mean #, SD, and Sources of Protein Servings Grouped According to Braden Nutrition Subscale Risk Categories (*n* = 690).

Protein Servings and Sources	Nutrition Risk: Very Poor (*n* = 25)	Nutrition Risk: Probably Adequate (*n* = 214)	Nutrition Risk: Adequate (*n* = 403)	Nutrition Risk: Excellent (*n* = 48)
	*Mean # (SD) per Meal*	*Mean # (SD) per Meal*	*Mean # (SD) per Meal*	*Mean # (SD) per Meal*
# Protein Servings	0.71 (0.32)	1.12 (0.52)	1.51 (0.54)	1.67 (0.42)
**Protein Sources:**				
Beans	0.06 (0.06)	0.11 (0.11)	0.17 (0.13)	0.19 (0.12)
Cheese	0.03 (0.03)	0.07 (0.07)	0.09 (0.08)	0.09 (0.07)
Egg	0.12 (0.07)	0.16 (0.09)	0.21 (0.09)	0.22 (0.09)
Lunch Meat	0.03 (0.03)	0.06 (0.05)	0.09 (0.07)	0.09 (0.06)
Meat	0.19 (0.10)	0.27 (0.14)	0.38 (0.17)	0.42 (0.15)
Milk	0.25 (0.15)	0.4 (0.21)	0.49 (0.22)	0.58 (0.22)
Nuts	0.03 (0.07)	0.04 (0.07)	0.04 (0.07)	0.04 (0.07)
Protein Powder	0.00 (0.01)	0.02 (0.05)	0.02 (0.07)	0.02 (0.09)
Soy	0.01 (0.01)	0.01 (0.04)	0.02 (0.05)	0.02 (0.03)

#### 3.2.3. Intake of Supplements and Snacks

Liquid supplements, either commercial or in facility formulations, were reported at some point during the study for 91% of residents. Those residents who were at high *versus* moderate PrU risk received supplements on a significantly greater percentage of study days (60.8 ± 34 *versus* 53.0 ± 34, *p* = 0.006). Snacks served by the facility or provided by families were given to 58% and 62% of high and moderate risk residents. Less than half of the snack was consumed by those who ate any part of the snack, with those at high risk for a PrU consuming significantly less than moderate risk residents (*p* = 0.01).

### 3.3. Braden Nutritional Risk and Dietary Adequacy

Statistically significant differences in weight (loss or gain) did not occur among residents, regardless of the nutrition subscale risk category assigned as part of the nursing staff’s PrU risk screening for all residents in the study. It was very rare for a resident in any nutrition risk category to experience a gain or loss in weight during the study (Table 4). Change in weight was most likely to occur in residents from the nutrition risk category 1—Very Poor with losses of 5%–10% and >10% being reported by 8% of residents (4% in each loss category). Similarly, the largest number of residents reporting weight gain was from nutrition subscale risk category 1—Very Poor, yet 4% of these residents had a 5%–10% gain in weight during the study period. Using change in weight as the outcome indicator of dietary adequacy, shows that although variation in % meal intake and # of protein servings per day was widespread, weight loss nor weight gain was not observed in 97.4% and 98.1% of residents, respectively. No change in weight was reported for any of the 10 residents who developed a PrU.

**Table 4:** Reported Weight Loss or Weight Gain Grouped According to Braden Nutrition Subscale Risk Categories (*n* = 690).

Braden Nutrition Risk Category	# In Risk Category	Weight Loss			Weight Gain		
		None	5%–10%	>10%	None	5%–10%	>10%
1 = Very poor	25	88.0%	8.0%	4.0%	96.0%	4.0%	0%
2 = Probably Adequate	214	97.2%	1.4%	1.4%	98.2%	0.9%	0.9%
3 = Adequate	403	97.8%	1.8%	0.5%	98.3%	1.7%	0%
4 = Excellent	48	100%	0%	0%	97.9%	2.1%	0%
	Total	97.4%	0.2%	0.01%	98.1%	0.02%	0.003%

## 4. Discussion

Dietary intake of NH residents in relation to PrU risk or PrU status (present/absent) has been reported previously in retrospective and prospective observational studies [1,2,3,4]. Many of these reports aimed to associate dietary intake and anthropometric or biologic markers of nutritional status with PrU development [4,5,6], or to determine differences between those with and without PrU [3,4,7]. More recently, interventions aimed at increasing dietary intake have been reported [5,23,24]. The residents of the TURN study [14] are fundamentally different than those in previously reported studies of nutrition and PrUs in that they are documented to be at moderate or high risk for PrUs (Braden Scale), have resided in the NH for 3 months prior to the study, and do not have a PrU at the outset of the study. Previous studies included all residents meeting age, length of stay, and PrU status criteria with the aim of identifying differences in those with PrUs, not always separating those who had PrUs on admission or developed PrUs during their residence in the facility. While there are methodological limitations in scope and specificity of study findings when using secondary data analysis, we believe further examining resident characteristics, nutrition risk, and dietary adequacy data from the randomized controlled trial significantly contribute to our clinical understanding about the utility of the nutrition subscale. The advantages of TURN study data include prospective observation of skin condition to confirm absence of a PrU at the outset of the study, ongoing observation by a nurse blinded to risk level, and daily dietary intake documented by CNAs. CNAs in NHs are responsible for recording the food eaten at each meal. Their training at the NH where employed is often supplemented with pictorials posted in break rooms with pictures of trays with various percentages of food eaten. Orientation to the study included a discussion of how to complete dietary intake forms as a means of minimizing variation and increasing fidelity of measures. As in most clinical studies there is the possibility of variation.

The Braden scale has two primary uses in the care of NH residents, one as a screening tool of resident’s overall risk for PrU development secondly to estimate the severity of risk factors, such as nutrition risk. Residents deemed to be at risk and at low risk for a PrU merit ongoing monitoring throughout their stay, since risk factors vary as overall health condition changes. Similarly, variation in nutrition risk occurs as changes in mobility, activity, and cognition change. Ratings for Braden subscales must be evaluated individually, in combination with each other, and in relation to the overall Braden Scale score in order to meaningfully use risk factor identification to effectively guide care planning. More specifically, this study adds to our understanding of the interrelationships between factors affecting nutrition risk and PrU development in NH residents. The Braden Scale served as a useful screening tool with which to gather information about overall risk for PrU development and information specific to the tool’s subscales. More specifically, we learned that when the 4 nutritional subscale risk categories comprising the Braden nutrition subscale are used for preliminary screening of nutrition risk, the subsequently observed mean meal intake for residents who were at either moderate or high risk for PrU development tended to approximate the intake defined for the respective category. We believe that the insights gained into a resident’s expected pattern of dietary intake suggest that the nutrition subscale may be a useful adjunct to more comprehensive nutritional screening. The presence of a nutritional diagnosis was rare among residents in this sample suggesting that early dietary intervention may not have been sought in the absence of initial nutritional screening through a mechanism such as the Braden Scale. Although not a comprehensive nutritional assessment, results in this study confirm that a meaningful connection exists between assessed nutrition subscale risk category and recent dietary intake and suggests that the Braden Scale’s utility extends beyond that of screening for PrU risk. Although this study did not aim to conduct a comparison of Braden subscale domains and nutritional risk, we believe that the Braden Scale has the potential for broader application by considering the influence of the various subscale domains on nutritional risk. This was especially true in this study, since mobility and activity subscale ratings were associated with nutritional risk for residents with a nutritional risk rating of 1—Very Poor. The results of this study clearly support the need for interpretation of all of the Braden Subscale ratings in combination to derive the greatest benefit from the screening evaluation and should serve as a basis of care planning.

Weight loss frequently occurs in NH residents with 9.9% prevalence according to Minimum Data Set quality reporting (≥5% body weight in 30 days) [25], is a negative indicator of resident condition, and is a trigger for feeding interventions. Liquid supplements and snacks are used frequently to prevent or treat residents with weight loss. In the study by Simmons and Patel [9] where supplementation was the focus of the study, 100% of the residents studied had an order for supplements, yet observations showed that 58% did not receive any supplements, and of those who were offered a supplement 16% did not consume the supplement. It was posited by Simmons and Patel that staff time, awareness of supplement orders, of supervision, and lack of specificity of orders may contribute to low delivery and ingestion of supplements [9]. Other studies also failed to show a benefit from supplementation, perhaps due to delayed utilization or inadequate amounts of supplement to make a difference [11]. Although residents studied by Simmons and Patel were demographically (gender, race, age, diagnoses) similar to TURN residents, specific data about the type of supplement, amount, and frequency of delivery is unknown for the TURN residents.

In this study, monitoring of dietary intake in conjunction with weight loss or gain as indicators of dietary adequacy proved valuable in further documenting the connections between nutrition risk category, intake, and sustainability of weight. The study period (21 days) was of a relatively short duration and may have been too short to detect significant weight change among those with little nutritional risk. However, residents dietary intake was observed for a long enough period of time to demonstrate that despite mean meal consumption of 82% for those in the nutrition subscale risk category 3—Adequate, the amount consumed was sufficient to sustain body weight. However, when mean intake was below 50% as for those in the nutrition subscale risk category 1—Very Poor, residents were prone to weight loss. Thus, identification of a resident as being in category 1, should signal the need for dietary consult as soon as possible. Even though snacks were given to slightly more than half of all residents studied, consumption of snacks was limited to less than half in most instances. The contribution that snacks were expected to make to overall dietary intake was not realized, raising questions about the efficacy of their use and suggesting the need for further study that explores strategies for making snacks appealing for consumption and examines how use of feeding assistance might lead to an increase in the amount consumed. Feeding assistance has been shown previously to increase the estimated daily intake and results in increasing or maintaining weight compared to a control group [10]; feeding assistance required on average 42 min for meals and 14 min/resident for snacks [5].

Protein intake is recognized for its role in helping to maintain skin integrity, thus preventing PrU development, and in its restorative capacity for fostering ulcer healing. All residents, regardless of PrU risk level or nutrition risk category, consumed far fewer protein servings per day than the 4 servings typically associated with dietary adequacy. Despite the ability for most residents to sustain weight during the study period, this shortfall in protein intake is of significant concern to overall health, especially if sustained over longer periods of time. Older adults are known to be susceptible to muscle wasting making adequate protein intake along with appropriate activity critical to their ability to maintain an optimal level of functional well-being.

Little definitive evidence exists to provide clear nutritional conclusions and recommendations for prevention of PrUs due to mixed findings and low quality studies despite the large amount of research concerning nutrition and PrU development. In a recent Cochrane Review [26], 11 randomized controlled trials tested medical nutrition therapy as an intervention to prevent PrUs. Overall findings were a lower incidence of PrU in control groups, but only one study’s findings reached statistical significance. In a meta-analysis of eight studies, there was no clear evidence to support nutritional supplementation in PrU prevention, concluding that it is unclear if nutritional supplementation reduces PrU development [26]. Although evidence is lacking to support specific nutritional interventions for PrU prevention, experts historically agree that nutrition should be included in the comprehensive care plan for prevention and management of PrUs [27]. In 2014, experts from the National Pressure Ulcer Advisory Panel (NPUAP), the European Pressure Ulcer Advisory Panel (EPUAP), and the Pan Pacific Pressure Injury Alliance (PPPIA) collaborative developed evidence-based recommendations for the prevention and treatment of PrUs that could be used worldwide [28]. The experts of this collaborative effort identified and critically appraised published scientific evidence, and determined specific recommendations in numerous categories for PrU prevention, including risk assessment, skin care, dressings, support surfaces, and medical devices [29].

Furthermore, the NPUAP/EPUAP/PPPIA practice guidelines include nutrition screening, nutrition assessment, care planning, energy intake, protein intake, hydration, and intake of vitamins and minerals [29]. Individuals at risk for a PrU or who have a PrU should be provided with a balanced diet that includes good sources of vitamins and minerals and receive vitamin and mineral supplements when nutritional deficiencies are present. It is also recommend that high-calorie, high-protein nutritional supplements should be offered to individuals whose meal intake is inadequate, while renal function is assessed and monitored along with encouragement of adequate daily fluid intake.

Upon admission and with each condition change, NPUAP/EPUAP/PPPIA practice guidelines recommend that a nutritional status screen and assessment should be performed with a valid and reliable nutrition and screening tool [29]. When nursing staff determine residents are at nutritional risk and/or have an existing PrU, the resident should be referred to a registered dietician (RD) or interprofessional nutrition team for comprehensive nutrition assessment. After nursing staff perform nutritional screening with a tool, such as the Braden Scale’s nutrition subscale, it is recommended that each resident be assessed from a nutritional perspective to include weight status, weight history, and determination of significant weight loss. The importance of taking into consideration the resident’s ability to eat independently and the adequacy of total nutrient intake is emphasized and consistent with the findings in this study.

The Braden Scale nutrition subscale is considered acceptable for use as a nutritional risk screening tool [27] and can be used for the first level nutrition screening described in the NPUAP/EPUAP/PPPIA practice guidelines, In addition to determining risk level, the usual food intake pattern component of the Braden Scale nutrition subscale offers clues to an individual’s hydration status and adequacy of total nutrient intake, including potential need for protein and nutrient supplementation. These data in conjunction with those from a more comprehensive nutrition assessment performed by the RD or interprofessional nutritional team serve as a valuable foundational resource in development of an individualized nutrition plan that helps to prevent PrU development and contributes to healing of existing PrUs.

## 5. Conclusions

Our ability to effectively plan for nutritional care based on identification of nutritional risk factors is inextricably linked to initial nutrition subscale screening, dietary intake, and individual outcomes (e.g., BMI and weight change). Furthermore, initial nutrition subscale risk ratings approximate subsequent dietary intake and, thus, can serve as a source of preliminary screening information that provides insight into meal intake patterns, especially when residents are challenged in their ability to consume meals. As evidenced by the association between nutrition, activity, and mobility subscale ratings and percent of meal intake, the Braden Subscale ratings should be evaluated in relation to the overall Braden Scale score and in combination with each to identify risk factors and use results as a basis for care planning. Further exploration of intake patterns and feeding assistance is needed in order to advance knowledge and evidence about successful nutritional interventions consistent with PrU prevention in residents at moderate and high risk for PrUs.
